# Vibrational Dynamics of Crystalline 4-Phenylbenzaldehyde from INS Spectra and Periodic DFT Calculations

**DOI:** 10.3390/molecules25061374

**Published:** 2020-03-18

**Authors:** Mariela M. Nolasco, Catarina F. Araujo, Pedro D. Vaz, Ana M. Amado, Paulo Ribeiro-Claro

**Affiliations:** 1 CICECO, Departamento de Química, Universidade de Aveiro, P-3810-193 Aveiro, Portugal; 2 Champalimaud Foundation, Champalimaud Centre for the Unknown, 1400-038 Lisboa, Portugal; 3 Química-Física Molecular, Departamento de Química, FCTUC, Universidade de Coimbra, P-3004-535 Coimbra, Portugal

**Keywords:** inelastic neutron scattering, density functional theory, vibrational assignment, molecular crystal, torsional potential, C-H···O hydrogen bonds

## Abstract

The present work emphasizes the value of periodic density functional theory (DFT) calculations in the assessment of the vibrational spectra of molecular crystals. Periodic calculations provide a nearly one-to-one match between the calculated and observed bands in the inelastic neutron scattering (INS) spectrum of crystalline 4-phenylbenzaldehyde, thus validating their assignment and correcting previous reports based on single molecule calculations. The calculations allow the unambiguous assignment of the phenyl torsional mode at ca. 118–128 cm^−1^, from which a phenyl torsional barrier of ca. 4000 cm^−1^ is derived, and the identification of the collective mode involving the antitranslational motion of CH···O bonded pairs, a hallmark vibrational mode of systems where C-H···O contacts are an important feature.

## 1. Introduction

The use of periodic density functional calculations (periodic DFT) to address the vibrational spectra of molecular crystals is becoming increasingly popular [1,2,3,4,5,6,7,8,9,10,11,12]. In fact, the periodic DFT approach, although originally developed to deal with extended inorganic systems, was found to be highly efficient in predicting the vibrational spectra of molecular crystals [13]. Periodic methods—available through programs such as VASP [14], CRYSTAL [15,16] and CASTEP [17]—are often used to assist vibrational assignments of infrared and inelastic neutron scattering (INS) spectra of molecular crystals [1,2,6,7,8,9,18]. Vibrational spectroscopy with neutrons provides information not accessible from the optical techniques (IR and Raman), due to the absence of selection rules. The relationship between periodic DFT calculations and INS spectroscopy is somewhat synergistic. The intensity of INS bands is proportional to the relative atomic displacements in normal modes [19], which, in turn, are a straightforward result of the computational approach to vibrational frequencies. The ability of periodic calculations to predict INS intensities with high accuracy leads to mostly unambiguous assignments of vibrational modes and, additionally, provides the means to test the validity of the molecular model used [6,20].

The crystal structure of 4-phenylbenzaldehyde has been addressed in a comprehensive crystallographic, spectroscopic and computational study of C-H···O hydrogen bonds [21]. More recently, the complete assignment of infrared and Raman spectra has been proposed based on discrete DFT calculations [22]. These works try to describe the vibrational spectra of the crystal through discrete calculations, either by choosing the simpler and most common “isolated molecule” approach [22] or by using several “dimer associations” to represent the possible interactions in the crystal [21]. Hence, 4-phenylbenzaldehyde is a suitable subject to test the “computational spectroscopy” approach to molecular crystals using periodic calculations. By combining periodic DFT calculations with INS spectra it is possible to validate assignments and assess vibrational dynamics of 4-phenylbenzaldehyde in the crystal.

## 2. Results and Discussion

### 2.1. Molecular Geometry and Intermolecular Interactions

**Figure 1:**
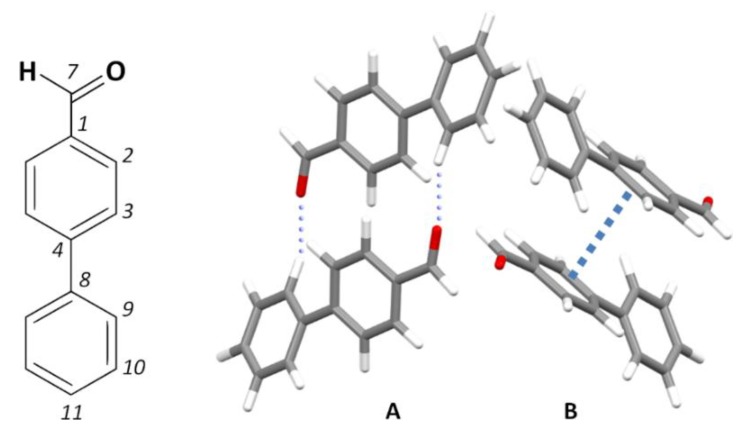
Representation of 4-phenylbenzaldehyde, with the atom labeling used throughout the text (left) and fragment of the crystal structure, evidencing the two types of dimer present in the crystal (right) [21].

Figure 1 presents a fragment of the crystal structure of 4-phenylbenzaldehyde, along with the numbering scheme adopted in this work. The crystal structure (monoclinic, space group P21/a, Z = 4) [21] is built from two types of dimers: the C-H···O bonded dimers (molecules labeled A) coexist with pi-stacking dimers (molecules labeled B), as shown in Figure 1. All molecules are involved in both types of dimers, but they have slightly different secondary neighboring contacts.

Table 1 compares some selected geometrical parameters obtained from X-ray [21], periodic calculations (CASTEP, see experimental details) and discrete single molecule calculations (G09, see experimental details). The “single molecule” model was chosen to perform the discrete calculations as it remains, despite its known limitations, a popular approach. The “dimer” model is not a straightforward option, due to the presence of different intermolecular contacts with identical strength (C-H···O and pi-stacking, dimers A and B in Figure 1). The alternative use of a “cluster” of molecules poses two main problems: the large number of molecules required to mimic the periodic crystal motif and the erratic behavior of the outer shell of the cluster (bound by weak intermolecular forces). In the past, a method based on the sum of pairwise contributions has been used as a less computationally demanding alternative to the cluster model [23,24,25] but with limited advantages.

There is a good agreement between experimental and calculated crystal structures (X-ray vs. CASTEP). The root mean square deviation between Cartesian coordinates of all atoms, excluding hydrogen atoms, is less than 5%, and RMS deviation in bond lengths, excluding C-H bonds, is less than 2%. Nevertheless, CASTEP geometrical parameters present some well-known limitations of PBE calculations. The C=O bond length is clearly overestimated—leading to the underestimation of the C=O stretching mode, already discussed elsewhere [2,8]—and the pi-stacking distance evidences the expected bias to large values: this distance would deviate to physically meaningless values if not constrained by cell dimensions [2].

The geometry of the single molecule obtained from discrete calculations (G09) at the PBE1PBE/6-311G(d,p) level (Table S1, Supplementary Materials) is in good agreement with the one recently reported at the B3LYP/6-311++G(d,p) level [22] and does not present large deviations from the CASTEP geometry. Table 1 also evidences the effect of crystal packing on the dihedral angles of aldehyde and phenyl groups. For the isolated molecule, the CHO group is predicted to be nearly coplanar with the ring, while the non-planarity between the two rings is heightened relative to the packed molecules.

### 2.2. Calculated vs. Experimental Spectra

**Figure 2:**
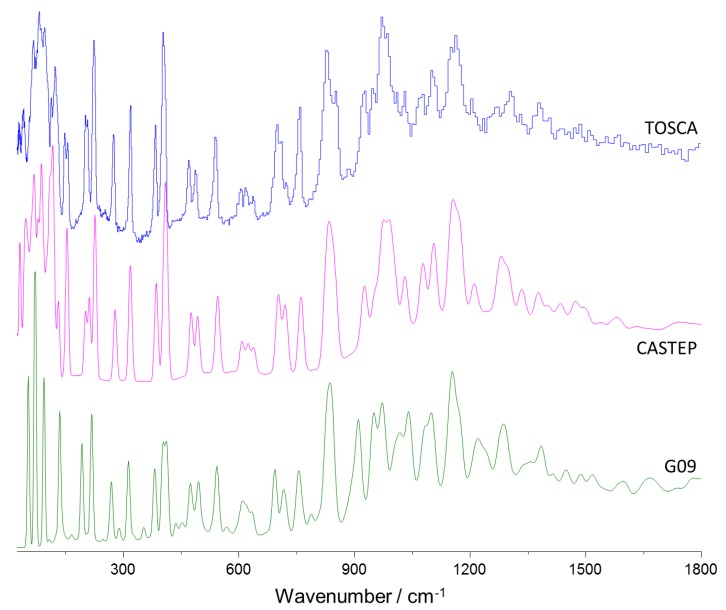
The inelastic neutron scattering (INS) spectra of 4-phenylbenzadhyde in the 25–1800 cm^−1^ range: experimental (top), simulated from periodic calculations (middle) and from single molecule discrete calculations (bottom).

Figure 2, top line, presents the experimental INS spectrum of 4-phenylbenzaldehyde (TOSCA), in the 25–1800 cm^−1^ range. Figure 2, middle and bottom lines, show the simulated INS spectra obtained from periodic (CASTEP) and discrete (G09) calculations, respectively.

The immediate impression from Figure 2 is the excellent agreement between the experimental spectrum and the one estimated from periodic calculations, with a remarkable nearly one-to-one correspondence between the bands, in both their positions and intensities. As for the spectrum simulated using the single molecule approach (Figure 2, bottom), the correlation was quite satisfactory for some regions, but with clear overall limitations. For instance, there were a few “extra” bands spread in the simulated spectrum, without correspondence in the experimental spectrum, and the description of the region 900–1200 cm^−1^ was poor. Additionally, of course, the calculated spectrum for a single molecule was meaningless in the region where the contributions from collective modes were expected to prevail (ca. <200 cm^−1^).

As stated above, the excellent description of the INS spectrum obtained from periodic calculations provided a straightforward validation of the model and allowed a trustful assignment of vibrational modes of crystalline 4-phenylbenzaldehyde. The full assignment of the INS bands based on the periodic calculations is presented in Table 2. The vibrational assignment of IR and Raman spectra of 4-phenylbenzaldehyde has been performed recently, based on discrete calculations at the B3LYP/6-311++G(d,p) level [22]. Although there is a general concordance between assignments, the INS spectrum allows the clarification of some spectral features, as discussed below.

The 4-phenylbenzaldehyde crystal presents 192 atoms in the crystallographic unit cell (24 atoms per isolated molecule) [21], from which 573 optical modes are predicted. These include 8 × 66 normal vibrational modes of the eight molecules (132Ag + 132Au + 132Bg + 132Bu) and 45 external modes describing translations and rotations (21 translational modes plus 24 librational modes, 12Ag + 11Au + 11Bg + 11Bu). 

**Figure 3:**
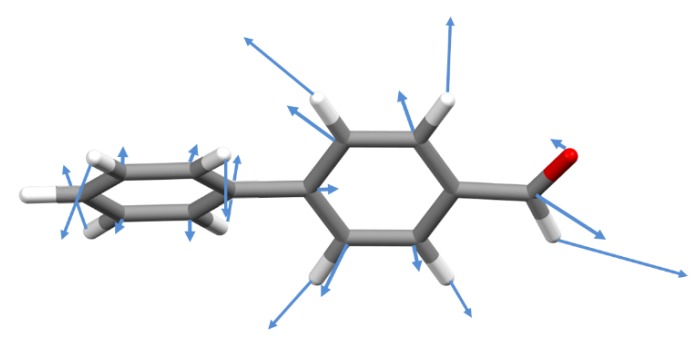
Atomic displacements of the normal mode at ca. 832 cm^−1^, assigned to a νC-C stretching mode of the benzaldehyde ring.

The 66 normal modes of vibration of each individual molecule include 60 related with the two phenyl rings. The “approximate description” of the normal modes in Table 2—obtained from the visualization of atomic displacements—is in accordance with the conventional notation for substituted aromatic rings: 23 stretching modes (labeled ν), 18 in-plane deformation modes (labeled αring and β) and 18 out-of-plane deformation modes (labeled δring and γ). In addition, there is one torsional inter-ring mode, (τ–C_6_H_5_) and six modes related to the aldehyde group (νC7=O, νC7-H, βC7=O, βC7-H, γC7=O and γC7-H, one of which is in fact the torsional mode, τ–CHO). This notation keeps the traditional separation between the “in-plane” and “out-of-plane” ring modes and assumes no significant interaction between internal and external modes. This latter assumption is expected to hold for systems with weak intermolecular binding [4], as it is the case of 4-phenylbenzaldehyde. However, the same is not true for the separation between “in-plane” and “out-of-plane” modes. Since the two aromatic rings are not coplanar, there is no molecular symmetry plane and the coupling between oscillators leads to normal modes of a hybrid nature. Figure 3 illustrates the case for the mode at ca. 832 cm^−1^. This mode is assigned to the νC-C stretching mode of the benzaldehyde ring (carbon and hydrogen atoms move in the same direction), but the contribution from the out-of-plane γCH modes is evident in the phenyl substituent ring (carbon and hydrogen atoms move in opposite directions).

The assignments in Table 2 can be compared with the ones recently reported [22]. The out-of-plane deformation mode of the aldehyde CH (γC7-H) was clearly assigned to the intense INS band at 990 cm^−1^, while the band at ca. 1030 cm^−1^ was ascribed to the ring CH bending modes (βC-H). The mode at 832 cm^−1^ was better described as ring stretching (νCC), despite its mixed nature, as described above. The contribution of ring deformation to the 623 cm^−1^ mode was high, but not more important than the C1-C7=O bond angle deformation, and thus the band was assigned to βC=O. This bending mode has been misassigned to the band at 475 cm^−1^ [22], which is, in fact, an out-of-plane deformation of the ring (δring). For the remaining low-wavenumber modes, the distinct description of the normal modes may arise from their complex mixture, which hampers the identification of the most relevant contribution in each case. This is an acknowledged limitation of the “approximate description” of the normal modes, chosen in favor of simplicity, as discussed elsewhere [2]. The exception is the inter-ring torsional mode (labeled phenyl torsion, τ-C_6_H_5_), which is unambiguously found to occur at much higher energy (ca. 109–115 cm^−1^) than predicted from discrete calculations.

### 2.3. Large Amplitude, Low Wavenumber Motions

**Figure 4:**
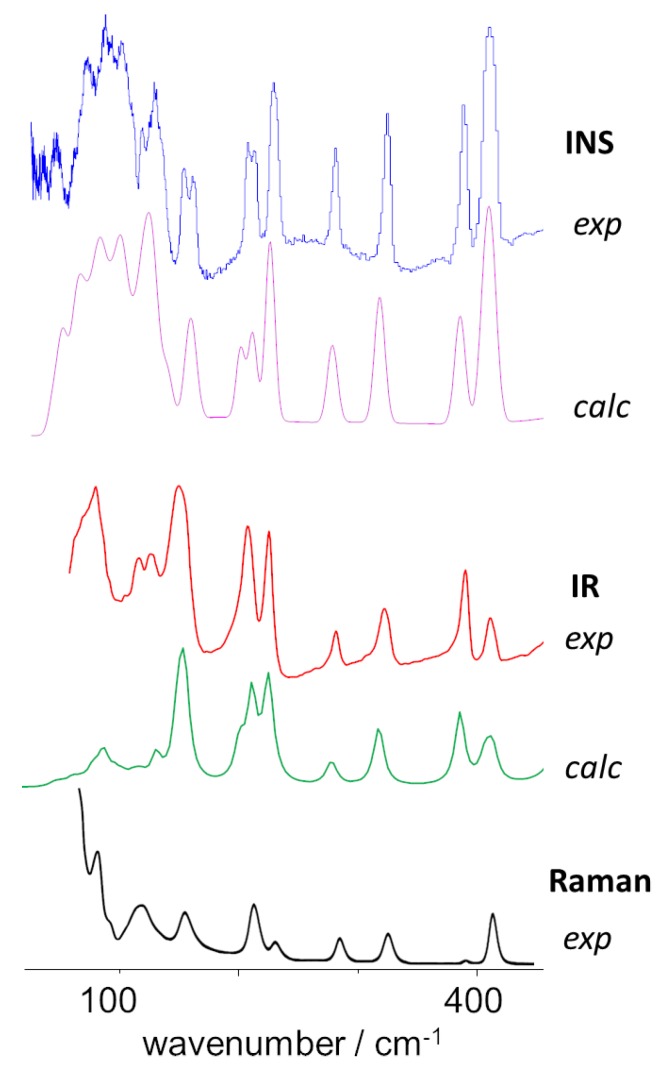
Vibrational spectra of 4-phenylbenzaldehyde in the spectral region below 450 cm^−1^. From top to bottom: INS spectra (experimental, calculated), far-infrared spectra (experimental, calculated) and Raman spectrum (experimental).

One of the major advantages of INS spectroscopy is to afford rich information in the low wavenumber region, providing a glimpse into the molecular dynamics of the systems. Both large amplitude molecular (internal) modes and crystal lattice collective (external) modes present a strong intensity in the INS spectrum, mainly due to the large displacements of hydrogen atoms. Figure 4 compares the Raman, IR and INS spectra o crystalline 4-phenylbenzaldehyde in the region below 450 cm^−1^, evidencing the richness of the INS spectrum.

As stated above, there is little interaction between internal and external modes in 4-phenylbenzaldehyde, which is consistent with a crystal packing based on C-H···O and pi-stacking weak interactions. In this way, it is still plausible to describe the large amplitude/low wavenumber modes in terms of internal vibrations of the molecules vs. collective motions.

Among the most relevant internal low-wavenumber modes there are the torsional motions of the aldehyde and phenyl groups (–CHO and C_6_H_5_–, respectively). The torsion of the –CHO group is predicted to be at ca. 148–153 cm^−1^ from periodic calculations (CASTEP) and at ca. 139 cm^−1^ from discrete calculations (G09). This latter value compares with the one obtained from similar discrete calculations at the B3LYP level (133 cm^−1^ [22]). 

As it can be seen in Figure 4, the calculated INS wavenumbers for –CHO torsion merge into a single band when assuming a reasonable linewidth. Nevertheless, the predicted band matches with the doublet found at ca. 152–162 cm^−1^, providing an unambiguous assignment. This value of the torsional frequency is above the value reported for the 4-fluorobenzaldehyde crystal (120 cm^−1^ [4]), and suggests a somewhat stronger restriction of the CHO group dynamics in 4-phenylbenzaldehyde.

The potential energy function for internal rotation is generally described by the sum of cosine terms,

(1) $$V\left(\theta \right)=\frac{1}{2}\underset{n}{\sum }V_{n}(1-\cos n\theta )$$

from which the energy levels for torsional motion can be derived by solving the Hamiltonian for the internal rotor. More usefully, the barrier height can be estimated by fitting the energy level differences to the observed torsional transitions [26].

**Figure 5:**
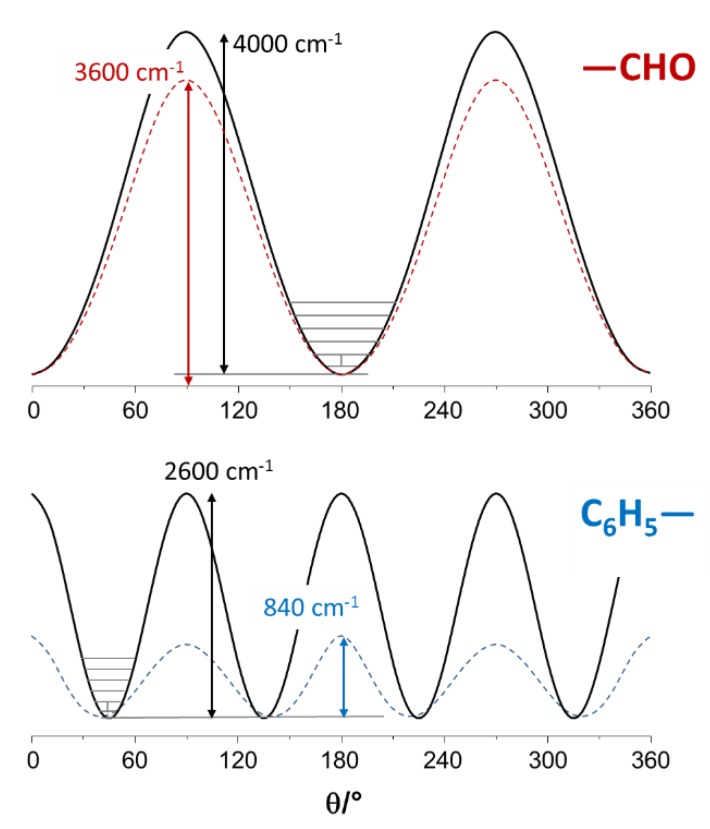
Potential curves for internal rotation of –CHO (top) and C_6_H_5_– (bottom) groups in 4-phenylbenzaldehyde. Dashed lines from single molecule calculations (G09, energy vs. dihedral angle), solid lines from experimental wavenumber in the crystal (Barrier [26]). The G09 potential curve for phenyl internal rotation (blue dashed line) is described by the terms V_2_ = −241 cm^−1^, V_4_ = 743 cm^−1^, V_6_ = 126 cm^−1^ and V_8_ = 51 cm^−1^.

For the isolated molecule, the internal rotation potential obtained from discrete calculations (G09) can be described by the V_2_ component, V_2_ = 3600 ± 100 cm^−1^ (42.6 ± 1.3 kJ/mol), consistent with the calculated torsional frequency of ca. 139 cm^−1^. This barrier height is in line with the one determined for benzaldehyde molecule from different quantum chemical calculations (ca. V_2_ = 2700–2900 cm^−1^) [27]. For the crystalline state, the experimental value of 153 cm^−1^ can be obtained from a V_2_ barrier of nearly 4000 ± 100 cm^−1^, (47.8 ± 1.3 kJ/mol, see Figure 5, top panel). Even though the assumption of a pure V_2_ barrier in the crystal is a crude approximation, it suggests that the internal rotation dynamics of the –CHO group is mainly determined by intramolecular properties. The intermolecular interactions resulting from crystal packing represent a modest barrier height increase of ca. 5.2 kJ/mol, which is consistent with the presence of weak CH···O interactions [21].

As for the torsion of the phenyl substituent (C_6_H_5_–), the effect of crystal packing is more pronounced. In fact, discrete calculations (G09) yield a torsional frequency value of only ca. 70 cm^−1^, while periodic calculations yield split torsional frequencies of 109–117 cm^−1^. Similar G09 calculations [22] lead to the assignment of phenyl torsion to a Raman band at ca. 65 cm^−1^. However, comparison between calculated and observed INS spectra in Figure 4 clearly suggests the assignment of the phenyl torsional mode in the crystal to the bands at ca. 118–128 cm^−1^, while the INS band at ca. 70 cm^−1^ is better ascribed to a collective “external” mode (Table 2). It should be mentioned that a phenyl torsion frequency of ca 116 cm^−1^ was recently identified in an ethylene terephthalate polymer (PET) from INS and CASTEP calculations [28].

The inter-ring torsional potential for the isolated molecule (G09) is dominated by a V_4_ component, with significant contributions from V_2_, V_6_ and V_8_ (Figure 5, bottom panel). However, the calculated torsional frequency of 70 cm^−1^ can be obtained from a simplified approach, assuming a pure V_4_ term of ca. 860 ± 30 cm^−1^ (10.3 ± 0.3 kJ/mol). Within the same approximation, the observed frequency of 128 cm^−1^ for the crystal corresponds to a V_4_ barrier of 2600 ± 80 cm^−1^. The large difference indicates that, in this case, the barrier to internal rotation is mainly dominated by the intermolecular contacts—most probably the steric hindrance resulting from crystal packing, which is expected to be significant for a large group such as the phenyl group. The reports on the torsional barrier for the phenyl group are scarce, but there is a tendency for a pronounced increase from the gas phase to the condensed phase, as shown in Table 3.

**Figure 6:**
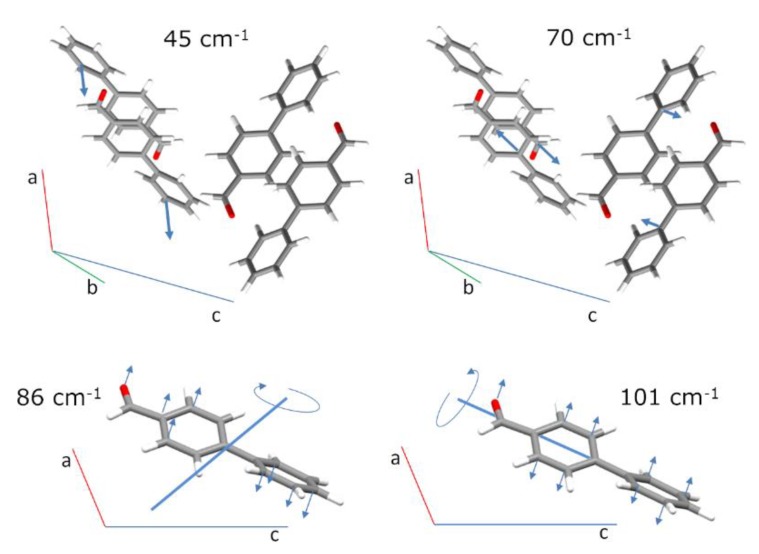
Schematic representation of atomic displacements for the external modes at 45 cm^−1^, 70 cm^−1^ (translations), 86 cm^−1^ and 101 cm^−1^ (librations). For better readability, only the total displacement of the molecules is shown for translations and only the displacements of heavy atoms are shown for librations.

The 45 external modes of the crystallographic unit cell give rise to a limited number of bands in the low wavenumber region. Table 2 identifies the four main bands observed in this region, and Figure 6 presents the atomic displacements of some external modes with relevant contributions to these bands. Of course, there are several other combinations of translational and librational motions accounting for the final intensities of the four bands. 

The translational mode at 70 cm^−1^ deserves particular discussion. As depicted in Figure 6, the full displacement of the molecules in this external mode results in the “slipping” between pi-stacked molecules, and the “antitranslational” motion of the CH···O bonded molecules. This antitranslational mode, which in fact represents the stretching vibration of the H···O intermolecular hydrogen bond (νH···O), has been identified in other systems through INS spectroscopy [33,34,35,36]. In the chloroform–acetone complex this mode has been ascribed to the INS band at ca. 82 cm^−1^ [33]. More interestingly, cyclopentanone dimers—with a symmetric double CH···O contact, as found for 4-phenylbenzaldehyde dimer—present the anti-translational mode at 95 cm^−1^ [34]. Although the mode in 4-phenylbenzaldehyde is not a pure hydrogen bond stretching, as it also involves the ring slipping of pi-stacking dimers, it falls in the expected wavenumber range for the CH···O interaction.

## 3. Materials and Methods

4-Phenylbenzaldehyde was obtained commercially (Sigma–Aldrich, CAS number 459-57-4) and used as supplied.

The ATR-FTIR spectra of solid 4-phenylbenzaldehyde (Figure S1, Supplementary Materials) was measured on a Vertex 70 spectrometer using a Platinum ATR single reflection diamond accessory. For the far-IR spectrum (50–600 cm^−1^) a silicon solid-state beam-splitter and a Deuterated L-alanine doped TriGlycine Sulphate (DLaTGS) detector with a polyethylene window was used. The mid-IR spectrum (400–4000 cm^−1^) was recorded using a Ge on KBr substrate beam-splitter and a liquid nitrogen cooled wide band mercury cadmium telluride (MCT) detector. All spectra were the average of two counts of 128 scans each. A spectral resolution of 2 cm^−1^ was used.

The Raman spectrum was recorded on a Jobin-Yvon T64000 spectrometer, in the subtractive mode configuration, using a 514.5 nm argon ion laser line, a non-intensified charge coupled device (CCD) detector, with an integration time of 5 s. The resolution was approximately 3 cm^−1^ and the estimated error in wavenumber was less than 1 cm^−1^. 

INS spectrum: the inelastic neutron scattering experiment was performed with the TOSCA spectrometer, an indirect geometry time-of-flight spectrometer at the ISIS Neutron and Muon Source at the Rutherford Appleton Laboratory (Chilton, UK). The sample, with a total amount of ca. 2 g, was packed inside a flat thin-walled aluminum can of 4.8 cm height and 4 cm width, with a path length of 2 mm, which was mounted perpendicular to the beam, using a regular TOSCA centre-stick. The spectrum was collected below 15 K, measured for the 16–8000 cm^−1^ energy-transfer range, and the resolution was ∆E/E ≈ 1.5%.

Quantum chemistry calculations: single molecule (discrete) calculations were performed with Gaussian 09 program (G09), version A.02 [37] using the PBE pure density functional combined with the 6-311G(d,p) basis set, as implemented in G09. Frequency calculations, subsequent to full geometry optimization, provide the infrared and Raman intensities and confirm the convergence to a true minimum (no imaginary frequencies). Potential energy functions for internal rotations have been obtained through the “scan” option (OPT = ModRedundant keyword) of G09, using the step size of 10°.

Periodic density functional theory (periodic-DFT) calculations were carried out using the plane-wave/pseudopotential method as implemented in the CASTEP code [17,38]. Exchange and correlation were approximated using the PBE functional [39]. The plane-wave cut-off energy was 830 eV. Brillouin zone sampling of electronic states was performed on the 8 × 4 × 4 Monkhorst-Pack grid.

The equilibrium structure, an essential prerequisite for lattice dynamics calculations was obtained by LBFGS geometry optimization after which the residual forces were converged to zero within 0.005 eV·A^−1^. The initial structure was taken from the reported crystal structure (CSD entry: WASLOS), and the cell parameters were kept constant during geometry optimization. This is important when using standard GGA functions, as their description of dispersion/van der Waals interactions is defective, leading to unrealistic cell dimensions. Phonon frequencies were obtained by the diagonalization of dynamical matrices computed using the density-functional perturbation theory. The eigenvectors (atomic displacements) for each normal mode that are part of the CASTEP output, enable visualization of the modes to aid assignments and are also all that is required to generate the INS spectrum using the program aClimax [40]. Program aClimax calculates INS intensities incorporating the instrumental bandwidth in a calculated spectrum that is easily compared with the experiment. It is emphasized that for all the calculated spectra shown the transition energies were not scaled.

Visualization of the atomic displacements of vibrational modes was performed using the Jmol program [41]. 

The determination of the potential barrier for a single simple internal rotor from torsional transitions has been performed with the program Barrier [26]. For an estimated error in torsional frequencies of less than 2 cm^−1^, the error in the barrier height was ca. 3%. 

## 4. Conclusions

The present work is a simple exercise that illustrates the capabilities of periodic DFT as an aid in the vibrational assignment of organic crystals. Despite being more resource intensive than its discrete counterparts, the effort of running periodic DFT calculations pays off by delivering accurate estimated spectra, which can be used as a direct guide for assignments. Even though 4-phenylbenzaldehyde individual units are held together by weak C-H···O and pi-stacking interactions—therefore a prime candidate for the “cheaper” discrete model approach—a periodic description is necessary for reproducing the full vibrational features. An example of the latter is the phenyl torsion, whose frequency is severely underestimated by the single molecule approach, misleading its assignment. From the corrected torsional frequency, the torsional barrier value of V_4_ = 2600 cm^−1^ was obtained for the phenyl group, well above the value of V_4_ = 860 cm^−1^ predicted for the isolated molecule. This contrasts with the torsional barrier of the aldehyde group, -CHO, which was clearly less affected by crystal packing (4000 cm^−1^ vs. 3600 cm^−1^, for the crystal and isolated molecule, respectively). In addition, periodic DFT is fundamental for understanding the low wavenumber region of INS spectra, offering a bird’s-eye view of collective modes such as the antitranslational motion of CH···O bonded pairs, a hallmark vibrational mode of systems where C-H···O contacts are an important feature.

## Figures and Tables

**Table 1:** Selected geometrical parameters of 4-phenylbenzaldehyde (molecules in dimers A and B).

	X-ray (A)	X-ray (B)	CASTEP(A)	CASTEP(B)	G09
*Bond length/pm*					
C7=O	118.1	118.7	122.7	122.8	120.6
C4-C8	149.2	145.5	147.8	147.8	147.7
C1-C7	148.7	148.4	146.8	146.8	147.4
*Dihedral angle/°*					
C2C1-C7O	−1.95	−2.06	−2.16	−2.26	−0.10
C3C4-C8C9	31.9	31.6	32.6	31.0	38.9
*Distance/pm*					
C9(H)···O	341	361	333.7	348.1	-
π...π (C1···C4’) ^1^	414.7	411.1	418.5	418.1	-

^1^ Distance between C1 and C4 atoms of the π-stacking molecules.

**Table 2:** Observed (TOSCA) and calculated (CASTEP) INS wavenumbers of 4-phenylbenzaldehyde.

CASTEP ^1^	Observed	Approx. Description
3104	3030	νC-H
1379	1384	βC7-H
1298	1312	βC-H
1170	1164	βC-H
1106	1108	βC-H
1077	1075	βC-H
1030	1032	βC-H
994	994	γC7-H
975	973	γC-H
953	951	γC-H
926	929	γC-H
845	855	γC-H
833	832	νC-C
762	762	γC-H
720	715	δring
702	702	δring
638	641	αring
622	623	βC=O
609	610	αring
545	545	δring
493	493	αring
476	475	δring
408	408	δring
385	388	βC8-C4
317	324	γC8-C4
278	281	αring
225	229	γC-CHO
210-201	212-207	βC-CHO
153	152-162	τ-CHO
130	136	βC4-C8
109-117	118-128	τ-C_6_H_5_
89	101	Libration (Ia)
67	86	Librations (Ic, Ib)
48	70	Translation (b,c)
37	45	Translation(a)

^1^ Maxima in the INS simulated spectrum. ν, α, β, γ and τ stand for stretching, in-plane deformation, out-of-plane deformation and torsion modes, respectively. Librations around the molecular axes of inertia, translations along cell axes.

**Table 3:** Comparison of V_4_ values for internal rotation of phenyl group derived from experiment.

System		V_4_/cm^−1^	Ref.
4-phenylbenzaldehyde	INS, crystal	2600 ± 80	This work
Biphenyl	Raman, crystal	2650–200	[29]
Anisole	Raman, crystal	4033	[30]
Trans-Stilbene	Fluorescence, supersonic jet	1550	[31]
4-Methoxy-stilbene	Fluorescence, supersonic jet	1430	[32]

## Supplementary Materials

The following are available online, Figure S1: Mid Infrared spectrum of 4-phenylbenzaldehyde in the 400–4000 cm−1 range (top), compared with the CASTEP calculated spectrum (bottom). Table S1: Coordinates of the optimized structure of 4-phenylbenzaldehyde (G09, PBEPBE/6-311G(d,p) keyword).
