# Supplementary material for: Vibrational Dynamics of Crystalline 4-Phenylbenzaldehyde from INS Spectra and Periodic DFT Calculations
Source: Molecules. 2020 Mar 18;25(6):1374. doi: 10.3390/molecules25061374 (PMC7144378; doi:10.3390/molecules25061374)
Supplement: Supplementary file 1 [file molecules-25-01374-s001.docx]

Vibrational Dynamics of crystalline 4-phenylbenzaldehyde from INS spectra and periodic DFT calculations

Mariela M. Nolasco ^1,^*, Catarina F. Araujo ^1^, Pedro D. Vaz ^2^, Ana M. Amado ^3^ and Paulo Ribeiro-Claro ^1^

**Supplementary Material**

**Figure S1.** Mid Infrared spectrum of 4-phenylbenzaldehyde in the 400–4000 cm^−1^ range (top), compared with the CASTEP calculated spectrum (bottom).

**Table S1.**  Coordinates of the optimized structure of 4-phenylbenzaldehyde (G09, PBEPBE/6-311G(d,p) keyword)


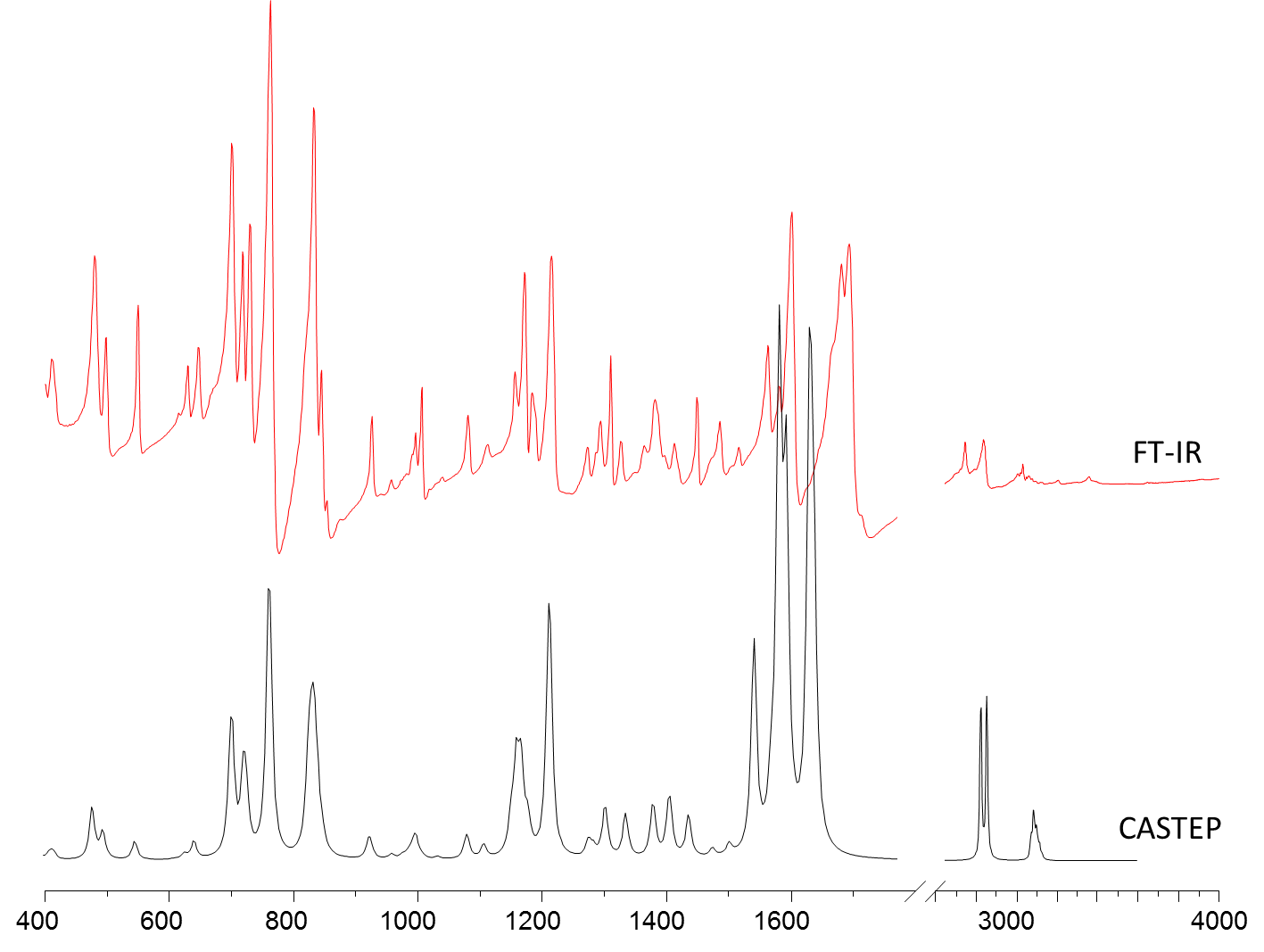


**Figure S1.** Mid Infrared spectrum of 4-phenylbenzaldehyde in the 400–4000 cm^−1^ range (top), compared with the CASTEP calculated spectrum (bottom).

**Table S1.**  Coordinates of the optimized structure of 4-phenylbenzaldehyde (G09, PBEPBE/6-311G(d,p) keyword)

6 -0.598147000 1.247170000 0.393181000

6 0.062468000 0.067869000 0.021695000

6 -1.984348000 1.306212000 0.408779000

1 -2.488063000 2.223576000 0.706394000

6 -0.710120000 -1.051229000 -0.332639000

6 -2.742048000 0.187887000 0.055621000

1 -0.210587000 -1.962275000 -0.649173000

6 -2.092872000 -0.994176000 -0.315862000

1 -2.699476000 -1.850347000 -0.595755000

1 -0.015570000 2.111772000 0.697171000

6 -4.214548000 0.254203000 0.074321000

8 -4.947912000 -0.665607000 -0.218467000

1 -4.629607000 1.238873000 0.385740000

6 1.538672000 0.002880000 0.002937000

6 2.210481000 -1.156559000 0.411532000

6 2.299865000 1.098887000 -0.423665000

6 3.599072000 -1.217302000 0.395633000

6 3.688364000 1.037008000 -0.441862000

6 4.343549000 -0.121047000 -0.031626000

1 1.638097000 -2.006799000 0.771644000

1 1.795701000 1.996255000 -0.771359000

1 4.101601000 -2.121889000 0.725628000

1 4.260500000 1.893882000 -0.785650000

1 5.428367000 -0.169010000 -0.045178000
